# Supplementary material for: Modeling glioblastoma heterogeneity as a dynamic network of cell states
Source: Mol Syst Biol. 2021 Sep 16;17(9):e10105. doi: 10.15252/msb.202010105 (PMC8444284; doi:10.15252/msb.202010105)
Supplement: Supplementary file 6 — Source Data for Figure 5 [file MSB-17-e10105-s004.zip › Figure5A_sourcedata/GSEA_3017/hallmarks_stateD.GseaPreranked.1621934542601/gsea_report_for_na_neg_1621934542601.html]

Report for na\_neg 1621934542601 [GSEA]

| GS  follow link to MSigDB | GS DETAILS | SIZE | ES | NES | NOM p-val | FDR q-val | FWER p-val | RANK AT MAX | LEADING EDGE || 1 | HALLMARK\_E2F\_TARGETS | Details ... | 102 | -0.41 | -2.17 | 0.000 | 0.009 | 0.008 | 558 | tags=70%, list=42%, signal=111% |
| 2 | HALLMARK\_G2M\_CHECKPOINT | Details ... | 77 | -0.41 | -2.15 | 0.000 | 0.007 | 0.012 | 322 | tags=47%, list=24%, signal=58% |
| 3 | HALLMARK\_APOPTOSIS | Details ... | 29 | -0.46 | -1.93 | 0.006 | 0.038 | 0.100 | 151 | tags=34%, list=11%, signal=38% |
| 4 | HALLMARK\_EPITHELIAL\_MESENCHYMAL\_TRANSITION | Details ... | 38 | -0.43 | -1.93 | 0.002 | 0.029 | 0.103 | 233 | tags=42%, list=18%, signal=50% |
| 5 | HALLMARK\_MITOTIC\_SPINDLE | Details ... | 59 | -0.36 | -1.77 | 0.007 | 0.079 | 0.315 | 520 | tags=59%, list=39%, signal=93% |
| 6 | HALLMARK\_GLYCOLYSIS | Details ... | 26 | -0.41 | -1.71 | 0.026 | 0.093 | 0.414 | 459 | tags=62%, list=35%, signal=92% |
| 7 | HALLMARK\_COMPLEMENT | Details ... | 15 | -0.49 | -1.64 | 0.040 | 0.126 | 0.559 | 115 | tags=33%, list=9%, signal=36% |
| 8 | HALLMARK\_SPERMATOGENESIS | Details ... | 19 | -0.42 | -1.59 | 0.042 | 0.146 | 0.674 | 252 | tags=42%, list=19%, signal=51% |
| 9 | HALLMARK\_TNFA\_SIGNALING\_VIA\_NFKB | Details ... | 29 | -0.36 | -1.55 | 0.053 | 0.164 | 0.761 | 213 | tags=31%, list=16%, signal=36% |
| 10 | HALLMARK\_ESTROGEN\_RESPONSE\_LATE | Details ... | 28 | -0.37 | -1.53 | 0.063 | 0.165 | 0.804 | 345 | tags=50%, list=26%, signal=66% |
| 11 | HALLMARK\_DNA\_REPAIR | Details ... | 21 | -0.39 | -1.53 | 0.060 | 0.154 | 0.812 | 344 | tags=52%, list=26%, signal=70% |
| 12 | HALLMARK\_UV\_RESPONSE\_DN | Details ... | 32 | -0.35 | -1.52 | 0.054 | 0.149 | 0.825 | 224 | tags=34%, list=17%, signal=40% |
| 13 | HALLMARK\_INFLAMMATORY\_RESPONSE | Details ... | 23 | -0.38 | -1.51 | 0.057 | 0.142 | 0.833 | 241 | tags=35%, list=18%, signal=42% |
| 14 | HALLMARK\_INTERFERON\_GAMMA\_RESPONSE | Details ... | 22 | -0.36 | -1.40 | 0.127 | 0.235 | 0.960 | 284 | tags=32%, list=21%, signal=40% |
| 15 | HALLMARK\_MYOGENESIS | Details ... | 28 | -0.33 | -1.36 | 0.132 | 0.266 | 0.983 | 325 | tags=39%, list=25%, signal=51% |
| 16 | HALLMARK\_IL2\_STAT5\_SIGNALING | Details ... | 23 | -0.34 | -1.34 | 0.148 | 0.268 | 0.989 | 82 | tags=22%, list=6%, signal=23% |
| 17 | HALLMARK\_MTORC1\_SIGNALING | Details ... | 34 | -0.30 | -1.32 | 0.152 | 0.270 | 0.992 | 163 | tags=24%, list=12%, signal=26% |
| 18 | HALLMARK\_HYPOXIA | Details ... | 35 | -0.30 | -1.31 | 0.162 | 0.271 | 0.996 | 607 | tags=66%, list=46%, signal=118% |
| 19 | HALLMARK\_P53\_PATHWAY | Details ... | 33 | -0.30 | -1.27 | 0.195 | 0.302 | 1.000 | 312 | tags=39%, list=24%, signal=50% |
| 20 | HALLMARK\_ADIPOGENESIS | Details ... | 18 | -0.32 | -1.16 | 0.310 | 0.419 | 1.000 | 18 | tags=11%, list=1%, signal=11% |
| 21 | HALLMARK\_APICAL\_JUNCTION |  | 20 | -0.28 | -1.07 | 0.382 | 0.519 | 1.000 | 718 | tags=75%, list=54%, signal=162% |
| 22 | HALLMARK\_ESTROGEN\_RESPONSE\_EARLY |  | 18 | -0.29 | -1.07 | 0.397 | 0.503 | 1.000 | 194 | tags=28%, list=15%, signal=32% |
| 23 | HALLMARK\_KRAS\_SIGNALING\_UP |  | 18 | -0.28 | -1.03 | 0.405 | 0.527 | 1.000 | 45 | tags=11%, list=3%, signal=11% |
| 24 | HALLMARK\_MYC\_TARGETS\_V1 |  | 26 | -0.24 | -1.00 | 0.474 | 0.558 | 1.000 | 180 | tags=23%, list=14%, signal=26% |
| 25 | HALLMARK\_OXIDATIVE\_PHOSPHORYLATION |  | 18 | -0.17 | -0.60 | 0.897 | 1.000 | 1.000 | 371 | tags=33%, list=28%, signal=46% |
| 26 | HALLMARK\_HEME\_METABOLISM |  | 18 | -0.14 | -0.53 | 0.939 | 1.000 | 1.000 | 949 | tags=83%, list=72%, signal=292% |
| 27 | HALLMARK\_UV\_RESPONSE\_UP |  | 16 | -0.15 | -0.51 | 0.949 | 0.991 | 1.000 | 747 | tags=69%, list=57%, signal=156% |
| 28 | HALLMARK\_XENOBIOTIC\_METABOLISM |  | 18 | -0.12 | -0.45 | 0.988 | 0.981 | 1.000 | 419 | tags=33%, list=32%, signal=48% |
Table: Gene sets enriched in phenotype **na **[plain text format]****
